# Effects of space allowance on patterns of activity in group-housed dairy calves

**DOI:** 10.3168/jdsc.2023-0486

**Published:** 2024-03-02

**Authors:** M.B. Ugarte Marin, K.N. Gingerich, J. Wang, H. Yu, E.K. Miller-Cushon

**Affiliations:** Department of Animal Sciences, University of Florida, Gainesville, FL 32611

## Abstract

With increasing use of social housing for dairy calves, there is a need to refine housing management practices that influence animal behavior and may affect welfare. Our aim was to assess the effect of pen space allowance on activity patterns and pen space use. Holstein heifer calves were group-housed (n = 6 pens; 5 calves/pen) at 14 d ± 2.8 d of age (mean ± SD). After a 7-d adaptation, each pen was exposed to 3 different space allowances (3.7, 4.6, and 5.6 m^2^/calf) in a random order, according to a replicated Latin square design with three 7-d periods (period 1, d 22–28; period 2, d 29–35; and period 3, d 36–42). Calves were provided milk replacer (12 L/d) ad libitum via an automated milk feeder and gradually weaned over 10 d, beginning at 48 ± 3 d of age. Using leg-based accelerometers (HOBO Pendant G data logger, Onset Computer Corp., Pocasset, MA), we obtained data describing standing time, standing bout frequency, and standing bout duration. Daily pen-level average standing time (6.5 h/d; SE = 0.27) did not differ between treatments. However, with greater space allowance, calves had more frequent standing bouts (22.6 vs. 20.3 bouts/d; 5.6 vs. 3.7 m^2^/calf; SE = 0.96) of shorter duration. To assess effects of space allowance on within-pen individual variability, we calculated the coefficient of variation for daily activity outcomes at the pen level and intra-class correlation coefficients for hourly standing time, by pen and day. The coefficient of variation for standing behavior outcomes decreased with increasing space allowance, and the intraclass correlation for hourly standing time increased, suggesting that increasing space allowance reduced individual variability and may promote more synchronous rest. Finally, we qualitatively assessed use of pen space using motion heat maps generated using computer vision from video recorded of each pen from 0800 to 1200 h on d 6 and 7 of each experimental period for each pen. These images suggest that calves preferentially used space near the perimeter of the pen, but space was used more uniformly when space allowance is restricted. Overall, these results suggest that lower space allowances may restrict patterns of activity at the pen level and reduce behavioral synchrony.

## Highlights

• Increasing space for dairy calves beyond industry practice affects activity.
• Providing group-housed calves with more space caused more frequent posture changes.
• Social synchrony in activity patterns increased with space allowance.
• More space may better accommodate preference for using specific pen areas.

## Graphical Abstract

**Figure:**
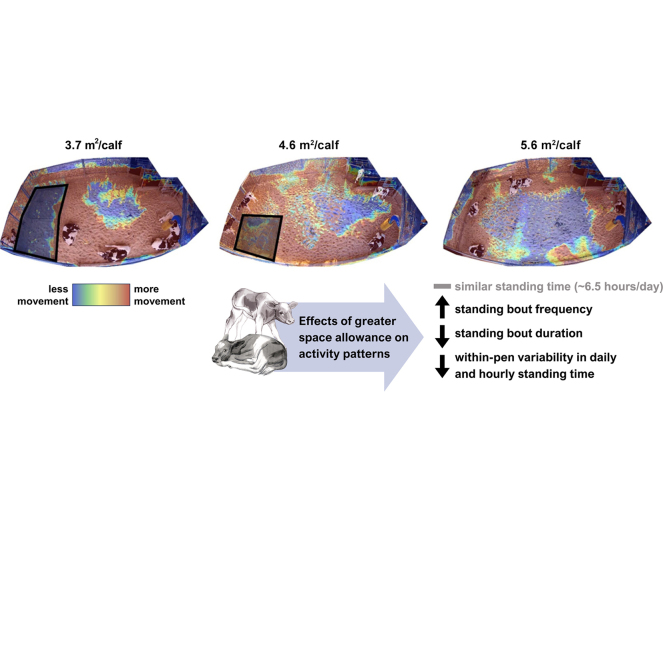
**Summary** We characterized how activity patterns of group-housed dairy calves are influenced by space allowances ranging from current industry-recommended minimum (3.7 m^2^/calf), to estimates of average on-farm space allowance in the United States (4.6 m^2^/calf), to more generous space (5.6 m^2^/calf). Using data collected from leg-based accelerometers, we found that pen-level daily average standing time was not affected by space allowance, but calves had more frequent and shorter standing bouts with increasing space. Withinpen variability in daily and hourly standing time decreased with increasing space, implying greater social synchrony when space was less constrained. Motion heat maps generated using computer vision illustrated that calves preferred to be near the edges of the pen, and restricting space caused more uniform usage of pen space. These results suggest that increasing space beyond current industry practice may allow for greater expression of individual behavioral preferences.

Social housing for dairy calves provides behavioral and performance benefits including stimulating greater solid feed intake and supporting the development of social behavior (Duve and Jensen, 2012; Miller-Cushon and DeVries, 2016). As social housing is increasingly adopted, there is a need to refine management practices that affect calf behavior and may have implications for welfare. A major housing factor affecting calf welfare is space allowance within the pen, which may affect calf health (Jorgensen et al., 2017) and has implications for behavioral expression (Sutherland et al., 2014; Jensen and Kyhn, 2000).

Access to freedom of movement and sufficient space for dairy cattle is of societal concern (Cardoso et al., 2016) and minimum space allowances for dairy calves are increasingly legislated worldwide. The EU requires space of at least 1.5 m^2^/calf (for group-housed calves <150 kg; Council of the European Union, 2008). While less prescriptive, state law in California requires that veal calves be able to “turn around freely” (California Department of Food and Agriculture, 2018). Recommendations for space allowance are greater than these minimum mandates (e.g., 2.8 m^2^/calf; ADSA, ASAS, and PSA, 2020; 3 m^2^/calf; EFSA Panel on Animal Health and Welfare, 2023), which appear to be reflected in on-farm practices for managing group-housed calves (e.g., mean 3.1 ± 1.89 m^2^/calf with groups of 11 ± 7.2 calves in a survey in Ireland; Barry et al., 2020, and 4.6 ± 2.0 m^2^/calf in groups of 17.6 ± 9.8 calves in a survey in Minnesota; Jorgensen et al., 2017).

When group-housed calves are provided more space, they play more (≥3 vs. ≤2.2 m^2^/calf; Jensen and Kyhn, 2000) and are more active, spending more time walking and standing, and less time lying (2 vs. <1.5 m^2^/calf; Sutherland et al., 2014), which may contribute to locomotor development. Further, individual variability in activity patterns and pen use may be subject to the space provided to group-housed calves, and lying space may affect synchrony of rest within a group of cattle (Færevik et al., 2008). Behavioral synchrony is considered a natural, motivated behavior of cattle, and it has been suggested that increased synchrony may imply improved animal welfare arising from sufficient resource availability (Stoye et al., 2012; Keeling et al., 2021). In group-housed dairy calves, much research to date has reported pen-wide averages in calf responses to housing factors, overlooking variability within the group, which may provide a more nuanced perspective on behavior and welfare.

The objective of this study was to assess effects of space allowances, representative of today's typical and increased space for group-housed calves, on activity patterns and within-pen variability in socially housed calves. We hypothesized that space allowance would alter activity patterns but we did not have specific predictions as to the direction of specific effects on standing time or standing bout frequency. We further predicted that space allowance would affect calves unequally, potentially increasing the within-pen variability in activity patterns and decreasing behavioral synchrony as stocking density increases. Our secondary objective was to descriptively examine pen use at different stocking densities. Although limited work to date has assessed how group-housed dairy calves use their space, some evidence of preference for secluded areas (e.g., see Gingerich et al., 2020) suggests that all space may not be perceived equally.

We enrolled 6 pens of group-housed Holstein heifer calves (5 calves/pen; 30 calves total) at the University of Florida Dairy Unit (Hague, FL). Calves were uniquely identified with radiofrequency identification ear tags and weighed at birth. Calves were initially housed in individual wire pens, allowing visual but not physical contact with other calves. Following colostrum bottle feeding (4 L within 6 h of birth), calves were provided 8 L/d of pasteurized waste milk via teat buckets in 2 daily feedings.

At approximately 2 wk of age (14 ± 2.8 d of age; mean ± SD), calves were moved to group pens (5 calves/pen). All calves within a pen were moved into the pen on the same calendar day, with an age range of 4.6 ± 1.4 d (mean ± SD) within each group. Birth weight (37.2 ± 7.0 kg; mean ± SD) did not differ between pens. Pens were deep bedded with sand and located under an open-sided barn (36.6 m long; 9.1 m wide) equipped with overhead fans for air circulation (no additional ventilation). The barn contained 8 group pens (3.7 × 8 m, with 28 m^2^ of available space), oriented in 2 rows of 4, with the same layout (varying orientation depending upon the location in the barn). Each pen contained one fence line at the exterior of the barn, and one at the interior of the barn. Fencing between pens was wire mesh, which permitted visual contact between adjacent pens. Each experimental group, as defined below, was housed in a different pen. All pens had ad libitum access to water and pelleted calf starter (22% CP and 2% fat; Ampli-Calf Starter Warm Weather, Purina Animal Nutrition LLC, Shoreview, MN) and 12 L/d calf milk replacer (28% CP and 15% fat with Bovatec and Clarifly; Southeast Milk Inc., Mayo, FL) fed through an automated milk feeder (DeLaval CF1000X, DeLaval, Kansas City, MO), which was located at a corner on the interior side of the pen. Calves received a weekly veterinary exam and were monitored daily for signs of illness by farm and research personnel. Only calves that were clinically healthy at the point of entry to group-housing were enrolled in the study.

After a 7-d adaptation to the group pen, during which all pens were provided 4.9 m^2^/calf, each pen (n = 6 pens/space allowance treatment; in line with Jensen and Kyhn, 2000; Sutherland et al., 2014) was exposed to different space allowance treatments of 5.6, 4.6, and 3.7 m^2^/calf in a random order, according to a replicated Latin square design with three 7-d periods (period 1, average 22–28 d of age; period 2, average 29–35 d of age; and period 3, average 36–42 d of age). Space allowance was adjusted by blocking areas within the pen using wire mesh pens (4.8 m^2^; none, 1, or 2), placed in the corner of the pen farthest from the autofeeder. These space allowances were selected to represent the current range from above average space for group-housed calves, recommended best practice, and accepted minimum requirements.

To visualize pen usage of calves on different space allowances, each pen was recorded by a digital video camera (Axis M2026-LE Network Camera, Axis Communications, Lund, Sweden) mounted in the center of the outside wall of the pen, approximately 3 m from the ground. For each group in each space allowance, we retrieved video recordings from 0800 to 1200 h on d 6 and 7 of the experimental period. During these days, there was minimum intervention from farm personnel, and only occasional presence of research personnel or veterinarians. For visually descriptive purposes, these data were utilized to generate motion heat maps to assess how space allowance affects calf movement and pen usage. We developed a Python pipeline to track and visualize movement patterns of dairy calves over time using the mixture of Gaussian (**MoG**) method implemented in the OpenCV package (Bradski, 2000). The pipeline works by first initializing an empty mask image based on the first input image. Then, the foreground objects are subtracted from the background using MoG and added to the mask image iteratively. After looping through all images, the accumulative foreground image is converted to a color-mapped image in a red, green, and blue color model and overlaid on the first input image to visualize the movement patterns.

Standing and lying time were measured continuously using electronic leg-based accelerometers (HOBO Pendant G data loggers, Onset Corp. Inc.; validated by Bonk et al., 2013), which were replaced every 2 wk. These data were extracted to calculate daily standing duration, daily standing bout frequency, daily standing bout duration, and hourly standing duration (UBC AWP, 2013). Daily measures of standing time (daily standing duration, standing bout frequency, and standing bout duration) were summarized by calculating (1) the pen-level average by day and (2) the CV (SD divided by the mean) to assess within-pen variability in activity at the day level. To assess within-pen similarity in activity at the hourly level, we calculated intraclass correlation coefficients (**ICC**) for hourly standing duration data, for each day and pen (measurements from 5 calves/pen for each hour). Variance estimates were generated from a 2-way random effects model (proc MIXED in SAS, v. 9.4. SAS Institute Inc.) and ICC were calculated as the ratio of the subject (i.e., hour) variance estimate, divided by the sum of the subject and error variance estimate (Liljequist et al., 2019). Values closer to 1 indicate complete agreement or consistency (i.e., similar values from each calf within the pen in the same hour), and lower values indicate less consistency. These data were analyzed in a general linear mixed model (proc MIXED in SAS) including fixed effects of space allowance treatment, day, treatment by day interaction, period, and treatment by period interaction (i.e., order of exposure to space allowance treatments), with pen as a random effect and day as a repeated measure. The variance-covariance matrix structure on the basis of best fit according to Schwarz's Bayesian information criterion (compound symmetry or autoregressive were selected for all data). Model residual plots were screened for normality. All values reported are LSM. Significance was declared at *P* < 0.05, and trends were reported if 0.05 ≤ *P* ≤ 0.10. No data were excluded from analysis.

Motion heat maps illustrating pen usage at different space allowances are shown for 3 example pens in Figure 1. Descriptive interpretation of these visualizations suggests that calves preferred to be near the edges of the pen and spent less time in the center of the pen when they had more space. In general, at the lowest space allowance, calves moved through the entire surface area of the pen more uniformly within the 4-h observation period used to create the motion heat maps. In contrast, there are larger areas at the center of the pen where there was less activity at greater space allowances.

**Figure 1:**
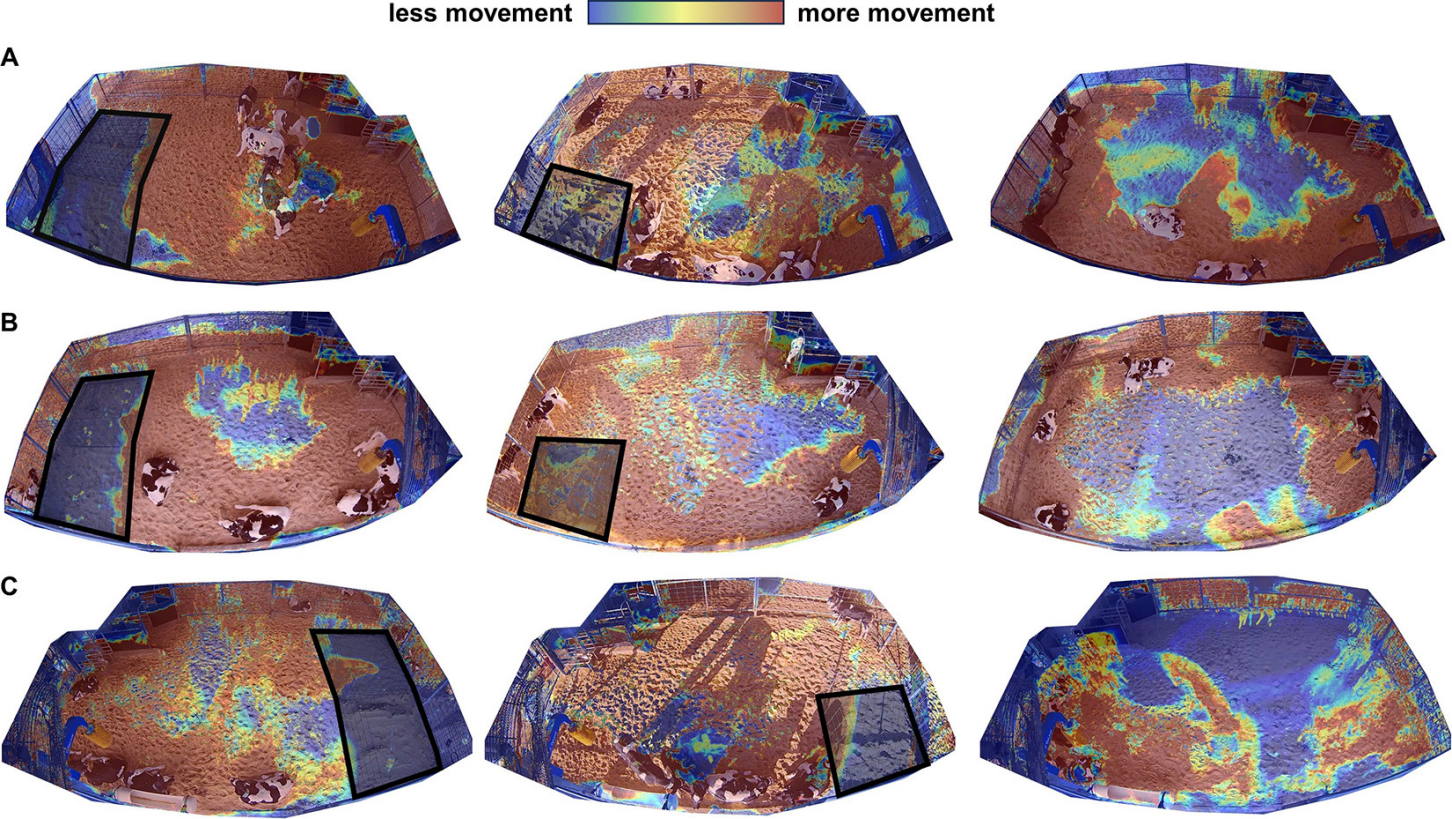
Motion heat maps illustrating use of pen space for 3 different group pens (A–C), with the 3 space allowances from left to right: 3.7, 4.6, and 5.6 m^2^/calf. The black box indicates the area that was blocked (using the addition of 1 or 2 wire mesh pens; 4.8 m^2^) to alter space allowance. Automated milk feeders are located in the upper right (A and B) or upper left (C) corners of the images. Troughs containing starter feed are located at the fence line at the bottom of each image. With increasing motion, areas are colored with warmer colors (red at areas of greatest motion), and areas with less motion are colored with cooler colors (blue at areas with no motion detected). Motion heat maps were generated based on cumulative motion from video recorded for 4 h (0800 to 1200 h) on d 6 and 7 of each experimental period for each group pen. The heat map image is overlaid on the first still image of each video (taken at 0800 h).

We found that daily pen-wide average duration of standing time was not affected by space allowance, but the frequency of standing bouts increased, and the duration of standing bouts decreased with greater space allowance (Table 1). These outcomes were not affected by day during the experimental period (*P* > 0.19), interactions between space allowance and day (*P* > 0.13), or experimental period (*P* > 0.21). With increasing space allowance, the within-pen CV for daily standing time decreased and within-pen ICC for hourly standing time increased (Table 1). Hourly patterns of standing time did not differ between space allowance treatments (no treatment by hour interaction; *P* = 0.8). Standing time is visualized for each pen and space allowance treatment in Figure 2, illustrating similar pen-level average daily and hourly duration of standing but greater within-pen variability (shaded regions denoting SD) at lower space allowances.

**Table 1:** Effects of space allowance^1^ on pen-level average activity patterns and within-pen variability^2^ (with 95% CI) for n = 6 pens (5 calves/pen), exposed to each space allowance for 7 d in a replicated Latin square design

Item	Space allowance			*F*_2,10_	*P*-value
	3.7 m^2^	4.6 m^2^	5.6 m^2^		
Pen-level average					
Standing time (h/d)	6.51	6.48	6.33	1.44	0.28
	(5.86, 7.15)	(5.84, 7.14)	(5.68, 6.97)		
Standing bout frequency (no./d)	20.6^a^	20.4^a^	22.5^b^	7.80	0.009
	(19.0, 22.1)	(18.9, 22.1)	(20.9, 24.1)		
Standing bout duration (min/bout)	20.3^a^	20.5^a^	17.7^b^	7.48	0.010
	(17.2, 23.2)	(17.5, 23.5)	(14.7, 20.7)		
Within-pen variability					
Standing time CV	17.4^a^	14.4^ab^	12.3^b^	6.44	0.016
	(14.5, 20.4)	(11.5, 17.4)	(9.4, 15.3)		
Standing bout frequency CV	23.7	18.8	21.1	3.03	0.093
	(19.5, 29.1)	(14.0, 23.6)	(16.3, 25.9)		
Standing bout duration CV	24.9	22.4	21.3	1.96	0.19
	(21.2, 29.0)	(18.4, 26.3)	(17.4, 25.2)		
Hourly standing time ICC	0.25^a^	0.38^b^	0.51^c^	25.7	<0.001
	(0.10, 0.40)	(0.24, 0.52)	(0.37, 0.65)		

a–c Values within a row with different superscripts differ (*P* < 0.05).

1 Space allowance treatments selected to represent the range from industry-recommended minimum space allowance (3.7 m^2^), typical average space allowance for larger groups of calves per survey results, and space allowance exceeding industry averages.

2 Within-pen variability was assessed using CV for daily standing time, standing, bout frequency, and standing bout duration (larger values reflect greater within-pen variability), and intraclass correlation coefficients (ICC) for hourly standing time by pen and day (larger values reflect greater consistency within the pen).

**Figure 2:**
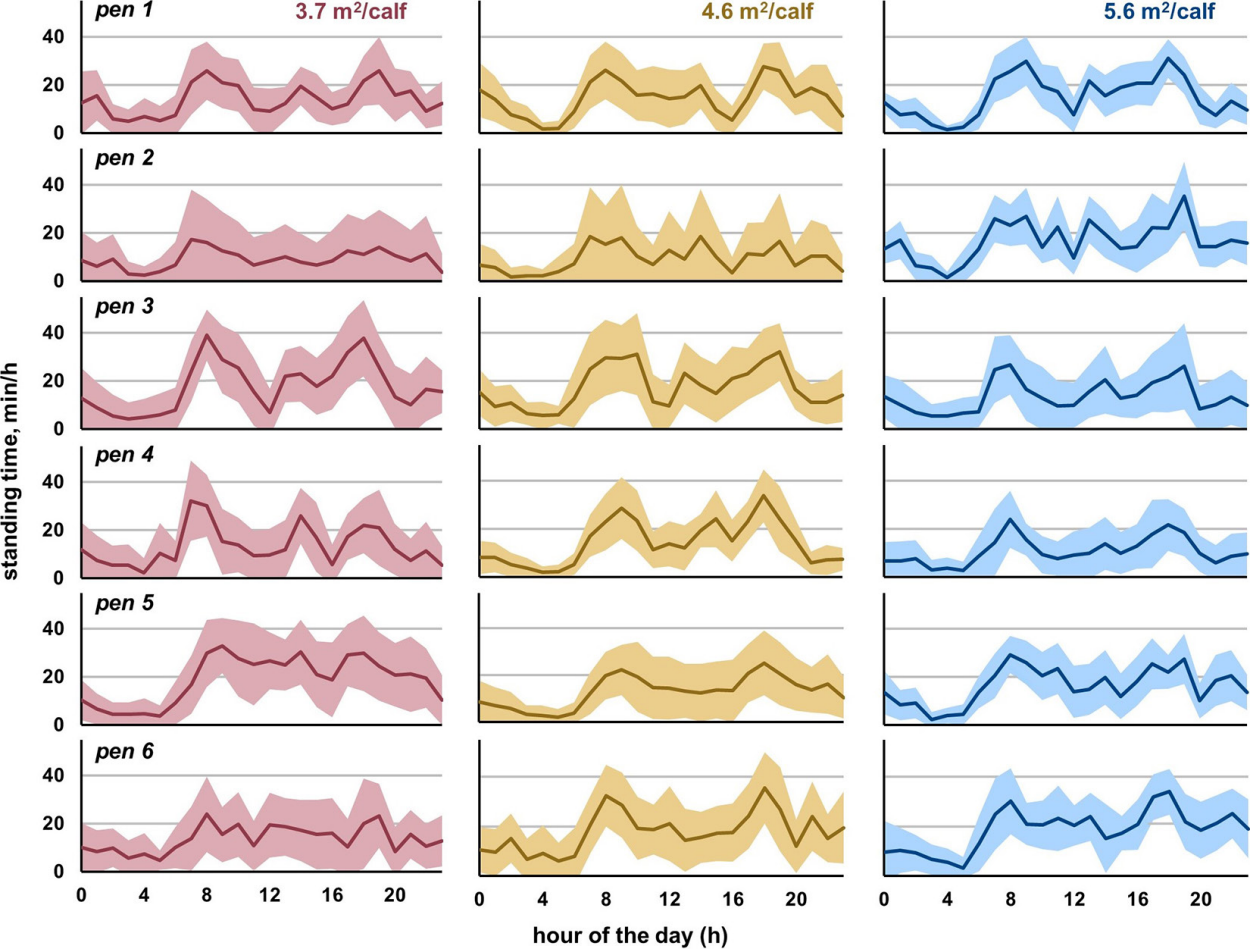
Hourly pen-level mean standing time (min/h; shown as a solid line) and within-pen SD (shaded region), averaged by pen (pen 1–6) and treatment (from left to right: 3.7, 4.6, and 5.6 m^2^/calf) across each experimental period (7 d of data collection).

Sufficient space for freedom of movement and the expression of natural behavior is widely viewed as a prerequisite for good animal welfare (Cardoso et al., 2016). In this study, we evaluated effects of space allowance on dairy calf activity, focusing on a range of space allowances meeting and exceeding current industry recommendations and standards for raising group-housed dairy calves.

Increasing space allowance beyond current industry recommendations resulted in a clear decrease in within-pen variability in daily duration of standing time and increase in similarity of hourly patterns of standing time. Observations of decreased synchronous rest coinciding with a reduced lying area have been previously noted in weaned dairy calves (Færevik et al., 2008) and older heifers (Nielsen et al., 1997). These results also align with effects of increasing stocking density on synchronous rest in adult cattle (Winckler et al., 2015). Behavioral synchrony has been proposed as a measure of positive welfare (Keeling et al., 2021), as it is viewed as a motivated behavior that may be constrained by housing design or resource availability. Our findings suggest that preferences for spatial distribution may not be accommodated at conventional space allowances for group-housed calves.

Given the lack of effect of space allowance on average daily standing time at the pen-level, our finding of increased variability within the pen implies that some individuals increased standing time while others decreased standing time as space per calf decreased. These findings complement a growing understanding that individuals respond differently to stressors. For example, dairy heifers vary in response to a regrouping event and individual degree of behavioral synchrony is related to competitive behavior (Nogues et al., 2020), and individual differences in how lactating cows respond to competition are related to personality traits (Schwanke et al., 2023).

We found that increasing space allowance did not affect pen-wide averages for standing time. Lower space allowances than those applied in the present study may be more likely to restrict preferred levels of activity, such as play (<3 m^2^/calf; Jensen and Kyhn, 2000) and walking and standing (<2 m^2^/calf; Sutherland et al., 2014). We did find that calves stood up and lay down more often when provided more space, particularly at 5.6 m^2^/calf compared with lower space allowances. Standing bout frequency is often interpreted based on context; for example, more frequent standing bouts may reflect exploration or restlessness following a housing change (Horvath and Miller-Cushon, 2018). In the present study, it is possible that more frequent posture movements may reflect greater opportunity for movement or access to pen spaces (e.g., repositioning within the pen) with greater space allowance.

To gain insight into pen space use, we applied a novel approach to generate a motion heat map using computer vision. Descriptive interpretation of these images suggests that there were pen-level preferences in regions of the pen, with calves spending more time near the edges of the pen. In an observational study of one group of dairy calves (26 calves; 2.4 m^2^/calf), Morita et al. (1999) similarly noted that calves avoided the pen center and preferred areas near feed and water and the wall, which was described as being used for lying. Although it is likely that preference for different pen areas may depend on a range of factors related to ventilation and pen design, these findings align with some consistent evidence of a preference in livestock for resting near the pen perimeter (Stricklin et al., 1979). This preference has implications for pen design and physical complexity; for example, Ehrlenbruch et al. (2010) found that goats spent more time resting near walls when provided additional walls. Although effects of physical structures on pen use in dairy calves have received only limited attention, some evidence suggests that group-housed calves may prefer resting in secluded regions at certain times; Gingerich et al. (2020) noted that some calves made considerable use of a 3-sided partition or ‘hide' and use increased following disbudding. As space allowance decreased, we found that pen use was more uniform, suggesting that preferences for peripheral pen areas (or avoidance of central pen areas) were less accommodated. These results describe a preliminary effort to use computer vision to descriptively characterize pen use and support further application of this developing technology to understand animal behavior and accommodate individual preferences in livestock housing environments. Future work may benefit from understanding individual animal movements as use of pen space may relate to individual traits, such as personality or coping style, and social dominance. For example, social dominance has been related to frequency of lying near a food source in loose-housed adult cattle (Nakanishi et al., 1992).

Findings from this study have implications for understanding calf activity patterns in future research. There has been considerable interest in characterizing how activity patterns relate to health status in group-housed dairy calves, yet studies to date report conflicting findings (reviewed by Costa et al., 2021). Our observed effects of space allowance on posture changes, individual variability, and synchrony of activity may explain inconsistencies in how activity patterns respond to disease, as well as other internal and external factors, across different studies. A limitation of this study is that it may not generalize to larger groups of calves seen on some commercial farms (e.g., dynamic groups of up to 60 calves fed by an automated milk feeder; Jorgensen et al., 2017). Like the present study, much behavioral research to date has been conducted with smaller pen replicates (e.g., 4 calves/pen; Jensen and Kyhn, 2000; Sutherland et al., 2014). Effects of management factors on behavior and performance of group-housed calves may well depend on group size, as increasing group size yields more effective space for a calf to move within but may increase competition.

In summary, our results suggest that increasing space allowance for group-housed dairy calves beyond current on-farm practice and accepted recommendations affected activity, by increasing standing bouts overall and reducing within-pen individual variability in standing patterns. These findings suggest that increased space may support synchronized patterns of rest, at preferred proximities or locations in the pen, and reduce individual variability. Our findings also suggest that individual calves respond differently to changing space allowances. We encourage further research to evaluate whether more generous space allowances may better accommodate individual differences and preferences, thereby improving animal welfare.
